# Attitudes, beliefs, and recommendations for persistent low back pain patients: cross-sectional surveys of students and faculty at a chiropractic college

**DOI:** 10.1186/s12998-024-00530-7

**Published:** 2024-02-29

**Authors:** Ryan D. Muller, Jesse Cooper, Jordan A. Gliedt, Katherine A. Pohlman

**Affiliations:** 1 VA Connecticut Healthcare System, West Haven, CT USA; 2 Research Center, Parker University , Dallas, TX USA; 3 Yale School of Medicine, Yale University, New Haven, CT USA; 4 Baylor Scott & White Health, Round Rock, TX USA; 5 Department of Neurosurgery, Medical College of Wisconsin, Milwaukee, WI USA

**Keywords:** Persistent low back Pain, Healthcare providers’ pain and impairment relationship scale, Activity and work recommendations, Chiropractic

## Abstract

**Background:**

While the use of chiropractic care for persistent low back pain (PLBP) is prevalent, chiropractors’ attitudes and beliefs related to PLBP patients are not fully understood. The purpose of this study was to assess the attitudes, beliefs and activity/work recommendations of students and faculty at a chiropractic college regarding PLBP patients.

**Methods:**

The Health Care Providers Pain and Impairment Relationship Scale (HC-PAIRS) and clinical vignettes were requested to be completed by chiropractic students and faculty at Parker University in April 2018. Higher HC-PAIRS scores indicate stronger beliefs that PLBP justifies disability and limitation of activities. Activity and work recommendations from clinical vignettes were rated as “adequate”, “neutral”, or “inadequate”, as defined in previous literature. Descriptive statistics, independent *t*-tests, and logistic regression were used to analyze results.

**Results:**

Student and faculty response rates were 63.6% and 25.9%, respectively. Faculty mean HC-PAIRS scores (3.66 [SD:0.88]) were significantly lower than students’ (4.41 [SD:0.71]). The percentage of faculty providing “adequate” activity (62.1%) and work (41.0%) recommendations was significantly greater than the percentage of students (activity: 33.9%, work: 21.2%) (*p* < 0.05). Higher HC-PAIRS scores in students were associated with decreased odds of providing “adequate” activity and work recommendations.

**Conclusions:**

Student and faculty attitudes and beliefs, and students’ activity/work recommendations were found to be dissimilar to those from similar studies and less congruent with CPG recommendations. Lower HC-PAIRS scores increased the odds of students providing “adequate” activity and work recommendations to patients with PLBP. Results from this study may help guide future research and training opportunities.

**Supplementary Information:**

The online version contains supplementary material available at 10.1186/s12998-024-00530-7.

## Introduction

Low back pain (LBP) is among the top ten most frequently seen conditions in primary care settings [1] and is associated with the highest overall cost of any condition in healthcare in the United States (US) [2]. LBP is the leading cause of disability globally [3] and in many cases, remains difficult to treat [4]. Disability and costs associated with LBP are expected to continue to rise in coming decades [4]. LBP research has recently demonstrated limitations in classifying the condition based on single time-points (i.e. acute, subacute, and chronic) and supports the use of terminology consistent with LBP trajectories (i.e. episodic and persistent low back pain– PLBP) [5].

Guideline-concordant care for LBP patients has demonstrated improved clinical outcomes and decreased costs associated with care [6–8]. Current clinical practice guidelines (CPGs) for LBP advise healthcare providers (HCPs) to recommend continuation of activity and early return to work, yet care for LBP is often not guideline-adherent [4, 9–11]. Evidence suggests HCPs with higher functional expectations regarding patients with PLBP are more likely to follow guidelines in clinical care [12–14], which may positively affect outcomes. Studies have also demonstrated that patients’ attitudes and beliefs about their pain are associated with their functional outcomes [15–17]. It is likely that HCPs’ beliefs regarding PLBP influences patients’ attitudes and beliefs about their own pain [18, 19]. Thus, HCPs’ attitudes and beliefs regarding PLBP patients may play an important role in potentially improving patient outcomes and decreasing costs.

Chiropractors have been found to have confidence in their abilities relating to treating PLBP [20]. Further, chiropractors serve as the first clinician seen for spine-related pain in up to 40% of patients in the United States (US) [21], as approximately 25% of individuals with persistent pain in the US seek chiropractic care [20]. Despite the prevalence of chiropractors’ confidence and engagement in the care of spine and persistent pain conditions, chiropractors’ attitudes and beliefs related to PLBP patients are not fully understood.

It has been speculated that students’ attitudes and beliefs regarding PLBP patients may persist into clinical practice and affect the way they manage patients in this population [22]. There has not been any assessment of the attitudes and beliefs, nor activity and work recommendations, of chiropractic students regarding PLBP patients. Therefore, it is important to measure chiropractic students’ attitudes towards PLBP patients throughout their training. In addition, student beliefs are likely influenced by the beliefs of their teaching faculty. Consequently, the attitudes and beliefs of faculty instructing these students should also be assessed.

The primary aim of this study was to assess the attitudes, beliefs, and activity/work recommendations of students and faculty of a chiropractic college regarding patients with PLBP. The secondary aim was to assess relationships between student and faculty attitudes and beliefs and activity and work recommendations.

## Methods

### Study design

This study is an analysis of a cross-sectional survey of chiropractic students and faculty at Parker University. This study was approved on 03/22/2018 by the Institutional Review Board of Parker University (Ref #A-00176). We reported this cross-sectional study following the Strengthening the Reporting of Observational Studies in Epidemiology (STROBE) guideline [23].

### Survey administration

All actively enrolled chiropractic students at Parker University were invited to participate in this cross-sectional survey in April 2018 (*n* = 781). The chiropractic program is organized into 10 terms, each lasting 15 weeks, with a total of 3 terms per academic year. This survey was presented during classes with an investigator inviting students to participate in the study. Students were given a QR code to the survey with time allocated to complete it. During the same time period, all faculty (*n* = 30) in the clinical sciences, chiropractic sciences, and student chiropractic clinic at Parker University were invited to complete this survey via a link sent in an email. To ensure anonymity, completion of the survey indicated consent to participate as stated on the first page of the survey, which was an informational letter. For sampling of students and faculty, neither group were given any extra theoretical or practical lessons on managing PLBP.

### Outcome measures

The survey instruments used were the Health Care Providers’ Pain and Impairment Relationship Scale (HC-PAIRS) and a series of three PLBP-related clinical vignettes. The HC-PAIRS is a 15-item measurement tool developed to assess the attitudes and beliefs of HCPs regarding functional expectations for patients with PLBP [18]. Higher scores on the HC-PAIRS indicate stronger beliefs that PLBP justifies disability and limitation of activities. The HC-PAIRS has been shown to be a valid and reliable assessment tool for HCPs using a 1–7 point rating scale (1 = completely disagree; 7 = completely agree), resulting in a theoretical score range of 15 to 90 [18, 24]. A 13-item HC-PAIRS questionnaire, with a theoretical score range of 15 to 78, also exists and is used in the literature [25]. We used the 15-item HC-PAIRS in the present study, and reported scores on a theoretical range of 1 to 7 (total score divided by the amount of items in the questionnaire) to allow for easier comparison of scores with other studies, regardless of whether the 13-item or 15-item tool was used. More recent literature has demonstrated that the HC-PAIRS measures a unidimensional construct, and that reporting scores per item and/or factor is unnecessary [25]. As such, we did not evaluate scores by factor or as single-items.

The three PLBP-related clinical vignettes used were assembled by Rainville et al. to explore physicians’ recommendations for work (1-full-time, full-duty; 5-remain out of work) and activity levels (1-no limitations on activity; 5- limit all physical activity) for PLBP patients [13]. Each scenario describes the patient’s symptoms, relevant physical findings, diagnostic test results, and previous treatment of patients who are out of work because of their LBP. Each scenario represents different degrees of severity, but none depicted evidence of structural damage or progressive neurological compromise that would require an operation. Activity and work recommendations given in the three clinical scenarios were classified as either “adequate”, “neutral”, or “inadequate” according to the convention established by Domenech, et al. [26] Activity recommendations of “no physical activity limitation” or “avoid painful activities” and work recommendations of “work full time at full duty” or “work full time at moderate duty” were considered to be “adequate”. “Limit activities to moderate exertion” and “work light duty, full-time” were considered “neutral”. “Limit activities to light exertion” or “limit all physical activities” and “work part-time with light duty” or “remain out of work” were considered “inadequate” recommendations for activity/work, respectively. The individual items of the HC-PAIRS and clinical vignettes are available as supplementary material.

### Data analysis

Descriptive statistics were calculated for participant demographic information (term, class size, sex, self-reported cumulative grade point average (GPA) on a 0.0 to 4.0 scale), total HC-PAIRS 15-item scores, and clinical vignette scores using Microsoft Excel and the Statistical Package for the Social Sciences (IBM SPSS, Inc., Version 28.0, Chicago, IL). Distribution frequencies were calculated for categorical variables, while means and standard deviations were calculated for numerical variables. Two-sample, two-sided independent *t-*tests were performed to compare the mean HC-PAIRS scores and activity and work recommendations of students and faculty.

We also evaluated the relationship between students’ HC-PAIRS scores and providing “adequate” activity and work recommendations using logistic regression. Students’ GPA and trimester were used as covariates in the regression. The relationships between faculty’s HC-PAIRS scores and activity and work recommendations were not assessed due to the small sample size of the faculty. Statistical significance was set as *p* < 0.05. Missing and/or incomplete data were not included in analyses.

## Results

Student and faculty response rates were 63.6% (*n* = 497) and 76.7% (*n* = 23), respectively. The average student grade point average (GPA) was 3.2 (SD: 0.50). The average number of years spent working at Parker University for faculty was 10.5 (SD: 9.47). Of faculty respondents, there were 8 (35%) from the Chiropractic Sciences Department, 9 (39%) from Clinical Sciences, and 6 (26%) who served as clinic faculty doctors in the outpatient student clinic.

### HC-PAIRS results

Mean HC-PAIRS scores for students and faculty were 4.41 (SD: 0.71) and 3.66 (SD: 0.88), respectively. The combined mean HC-PAIRS score for students and faculty was 4.38 (SD: 0.73). Faculty mean HC-PAIRS scores were significantly lower than those of students (*p* < 0.001).

**Fig. 1:**
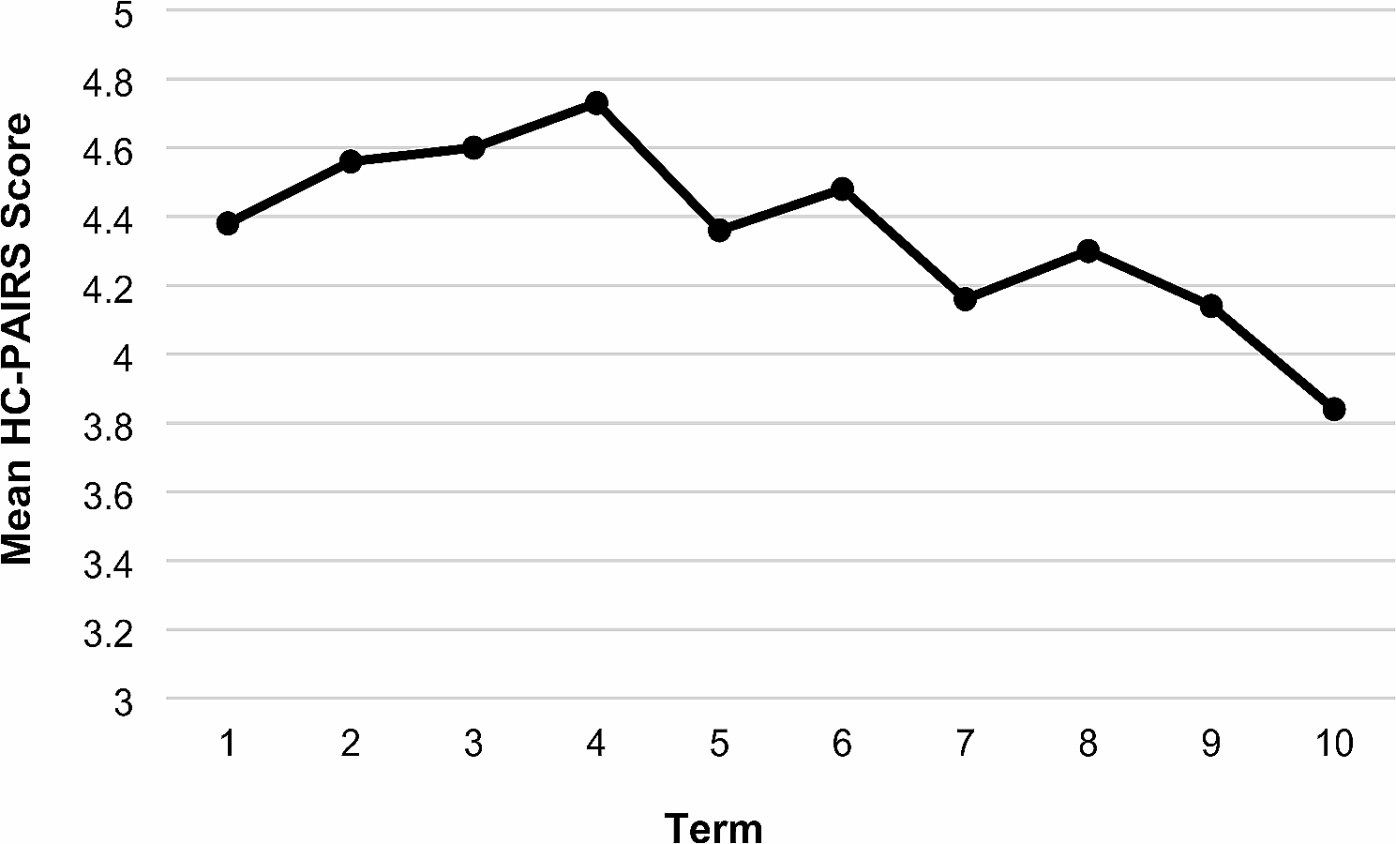
Students’ Mean HC-PAIRS Scores by Term. HC-PAIRS: Healthcare Providers Pain and Impairment Relationship Scale. The solid black line represents scores in 2018, while the dotted black line represents scores in 2020

Student demographics and mean HC-PAIRS scores by term are shown in Table 1. Students’ mean HC-PAIRS scores by term are displayed in Fig. 1. Overall, mean HC-PAIRS scores tended to be lower in students in later terms compared to students in earlier terms. Mean HC-PAIRS scores were lowest in term 10.

**Table 1:** 2018 student demographics and mean HC-PAIRS Scores

	n	Class Size	Response Rate	% Male	Mean Cum. GPA	Mean HC-PAIRS Score (SD)	HC-PAIRS Range (Min, Max)
Term 1	73	83	87.95%	45.2	3.38 (SD: 0.60)	4.38 (0.63)	3.80, 6.13
Term 2	106	124	85.48%	51.9	3.26 (SD: 0.44)	4.56 (0.64)	3.73, 6.40
Term 3	53	63	84.13%	64.2	3.27 (SD: 0.61)	4.60 (0.73)	3.67, 6.40
Term 4	31	59	52.54%	61.3	3.02 (SD: 0.36)	4.73 (0.77)	3.80, 6.33
Term 5	45	83	54.22%	57.8	3.21 (SD: 0.36)	4.36 (0.71)	3.40, 5.93
Term 6	32	61	52.46%	50.0	2.86 (SD: 0.31)	4.48 (0.60)	4.07, 6.07
Term 7	57	78	73.08%	59.7	3.16 (SD: 0.38)	4.16 (0.68)	3.40, 6.40
Term 8	59	95	62.11%	57.6	3.21 (SD: 0.41)	4.30 (0.70)	2.73, 6.33
Term 9	29	47	61.70%	55.2	2.97 (SD: 0.70)	4.14 (0.74)	3.20, 5.93
Term 10	12	88	13.64%	58.3	3.08 (SD: 0.40)	3.84 (0.78)	2.73, 5.33
Missing or incomplete– N (%)	-	-	-	20 (4.0)	41 (8.2)	23 (4.6)	-
Overall	497	781	63.64%	55.9	3.2 (SD: 0.50)	4.41 (0.71)	2.73, 6.40

Avg: average; %: percentage; Cum: cumulative; GPA: grade point average; HC-PAIRS: Healthcare Providers Pain and Impairment Relationship Scale; SD: standard deviation.

### Clinical vignette results

**Fig. 2:**
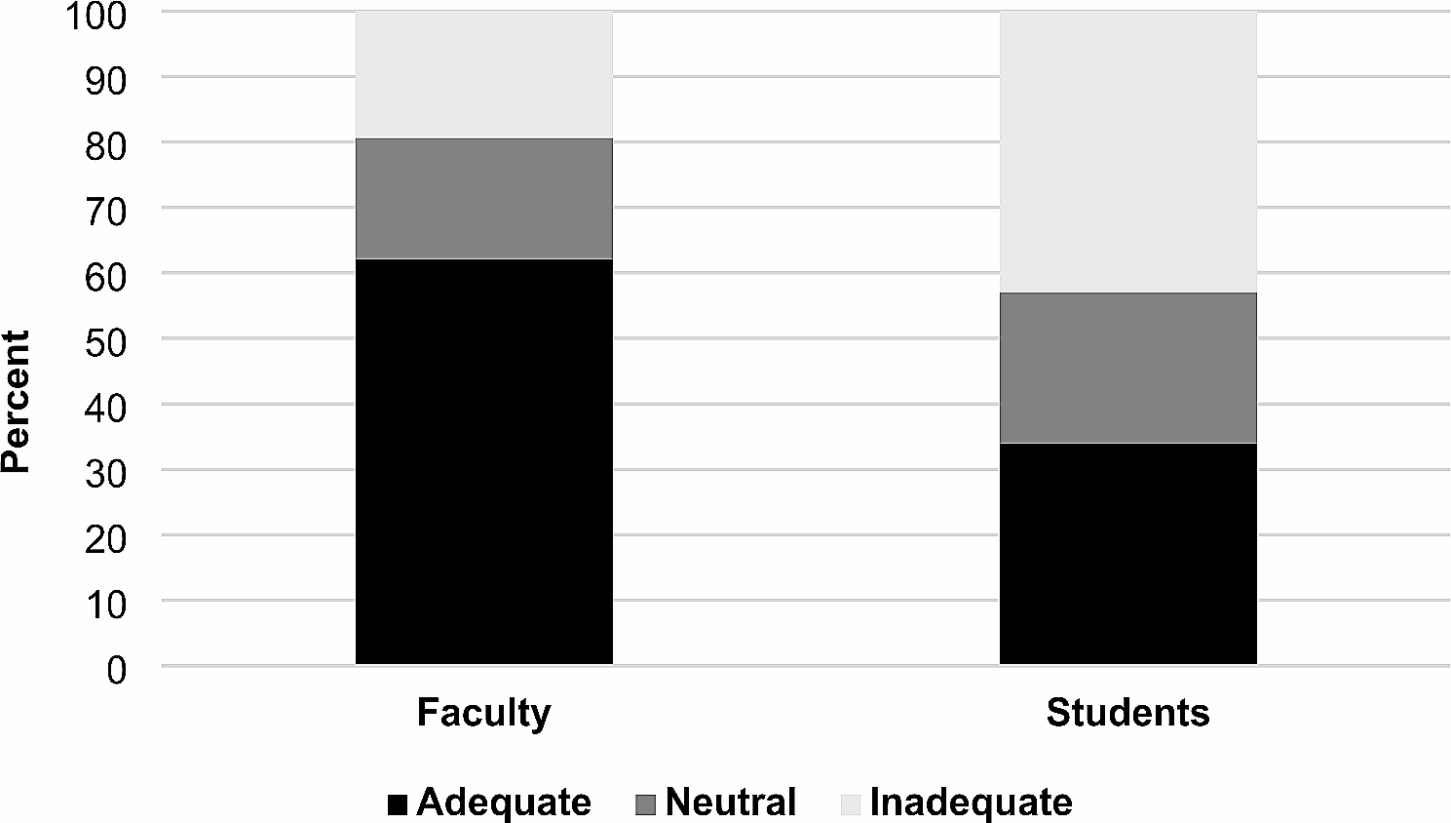
Mean percentage of students and faculty providing adequate, neutral, and inadequate activity recommendations. Black shading represents the percentage of adequate, dark gray represents the percentage of neutral, and light gray represents the percentage of inadequate activity recommendations given for each population

**Fig. 3:**
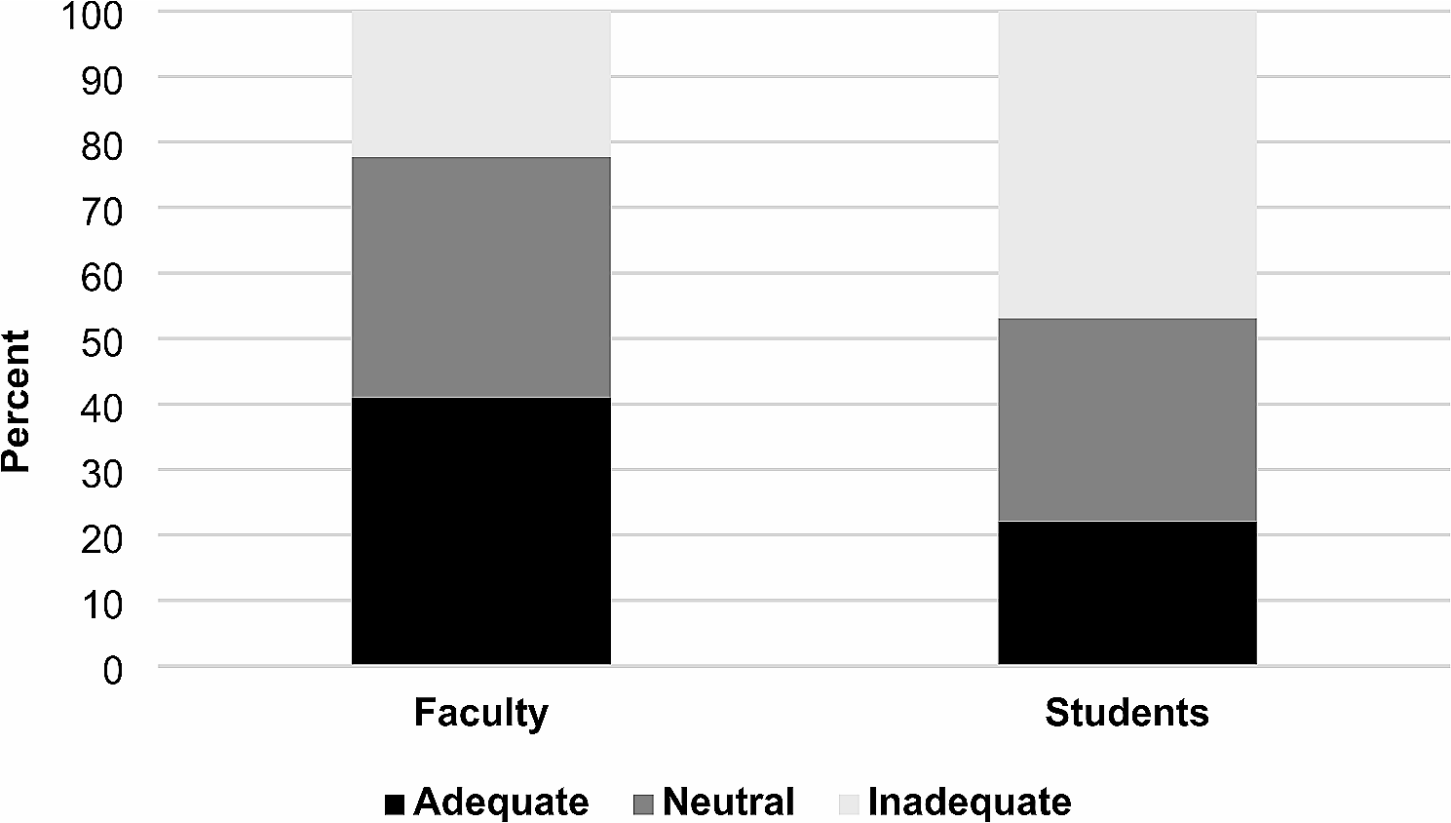
Mean percentage of students and faculty providing adequate, neutral, and inadequate work recommendations. Black shading represents the percentage of adequate, dark gray represents the percentage of neutral, and light gray represents the percentage of inadequate activity recommendations given for each population

The results of the clinical vignettes regarding activity/work recommendations are shown in Figs. 2 and 3. The percentage of faculty providing “adequate” activity (62.1%) and work (41.0%) recommendations was significantly greater than the percentage of students (activity: 33.9%, work: 21.2%) providing “adequate” recommendations (*p* < 0.05). Missing or incomplete data regarding both activity and work recommendations as measured by clinical vignettes were present in 24 (4.8%) respondents.

### Relationship between students’ HC-PAIRS scores and clinical vignette recommendations

Students who had lower HC-PAIRS scores (indicating higher functional expectations for patients with PLBP) were found to be significantly more likely to provide both “adequate” activity and work recommendations (Table 2). These results suggest that for every one-point increase in HC-PAIRS score (indicating lower functional expectations), students are 48% less likely to give “adequate” activity recommendations and 61% less likely to give adequate work recommendations to patients with PLBP.

**Table 2:** Relationship between students’ HC-PAIRS scores and activity/work recommendations

Variable	Provide adequate activity recommendations				Provide adequate work recommendations			
	OR	Std. err.	*p*-value	95% CI	OR	Std. err.	*p*-value	95% CI
HC-PAIRS score	0.519	0.111	0.002	0.341, 0.790	0.392	0.160	0.021	0.176, 0.870
GPA*	0.991	0.068	0.898	0.867, 1.133	0.850	0.413	0.739	0.328, 2.205
Trimester* (ref: 1)								
2	1.911	1.181	0.294	0.570, 6.415	0.738	0.752	0.766	0.100, 5.435
3	4.362	2.749	0.019	1.268, 14.999	0.729	0.908	0.800	0.064, 8.360
4	3.136	2.367	0.130	0.714, 13.771	1.439	1.806	0.772	0.123, 16.843
5	3.483	2.276	0.056	0.968, 12.537	0.678	0.849	0.756	0.058, 7.885
6	3.267	2.342	0.099	0.802, 13.313	1.106	1.391	0.936	0.094, 12.997
7	2.344	1.522	0.190	0.656, 8.375	0.461	0.576	0.535	0.040, 5.338
8	3.613	2.325	0.046	1.023, 12.759	2.596	2.347	0.291	0.441, 15.274
9	1.089	0.989	0.925	0.184, 6.461	1.0	-	-	-
10	4.061	3.520	0.106	0.743, 22.198	4.090	4.533	0.204	0.466, 35.906

HC-PAIRS: Healthcare Providers Pain and Impairment Relationship Scale; OR: odds ratio; CI: confidence interval; Std. err.: standard error; GPA: grade point average; *: variable used as covariate in logistic regression model; ref: reference category in logistic regression; -: empty, no respondents in Trimester 9 provided adequate work recommendations

## Discussion

This study assessed the attitudes and beliefs regarding PLBP patients of students and faculty at a single chiropractic college. Faculty mean HC-PAIRS scores (3.66 [SD: 0.88]) were more consistent with clinical practice guidelines (CPGs) [27] than those of students (4.41 [0.71]). This suggests that faculty in this study held higher functional expectations for patients with PLBP compared to students. This study also found mean HC-PAIRS scores tended to be lower (indicating stronger beliefs that PLBP does not justify disability and limitation of activities) in students in later terms compared to students in earlier terms, which has been observed in similar studies [28]. In addition, on average, faculty provided more “adequate” activity (62.1%) and work (41.0%) recommendations for patients with PLBP compared to students (activity: 33.9%; work: 22.1%) (*p* < 0.05). Students with lower HC-PAIRS scores (indicating higher functional expectations for patients with PLBP) were significantly more likely to provide “adequate” activity and work recommendations than students with higher scores.

**Fig. 4:**
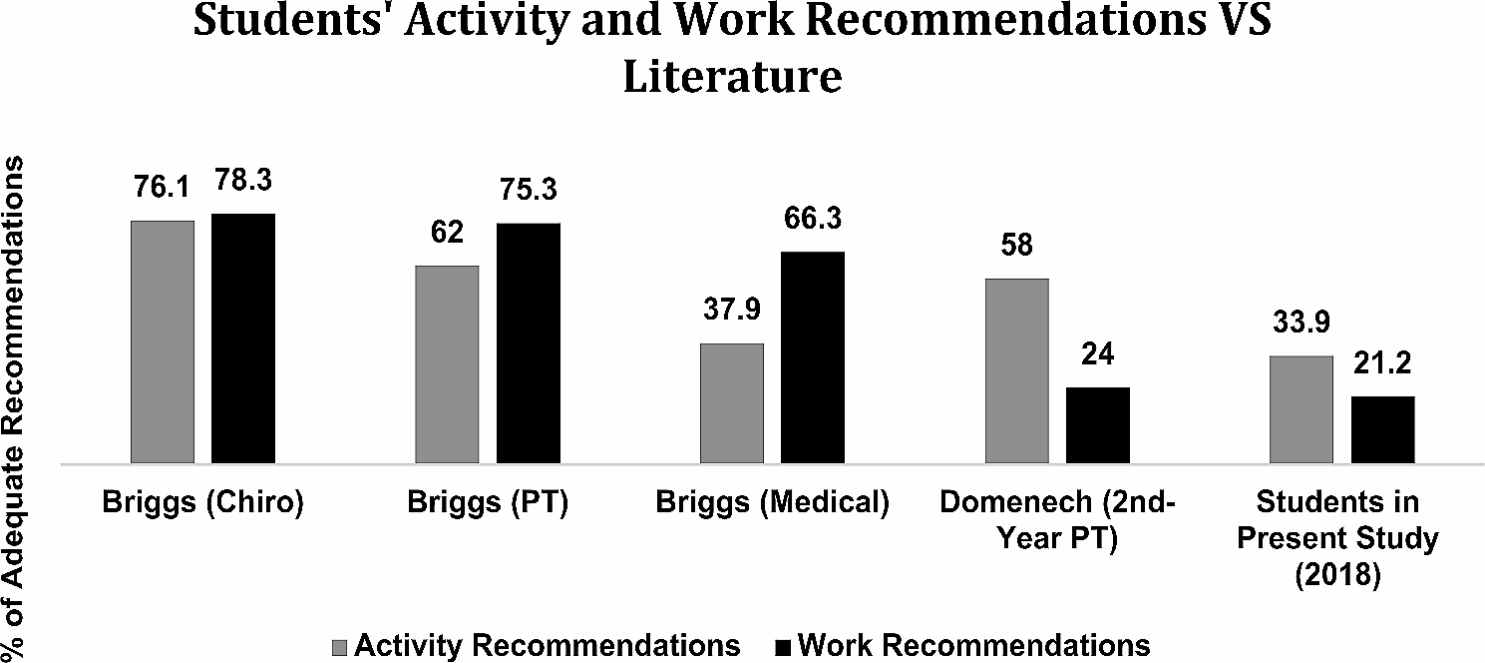
Students’ activity and work recommendations vs. literature [26, 29]. Chiro: chiropractors; PT: physical therapists; Medical: medical doctors. Gray shading represents the mean percentage of students that gave adequate activity recommendations. Black shading represents the mean percentage of students that gave adequate work recommendations

**Fig. 5:**
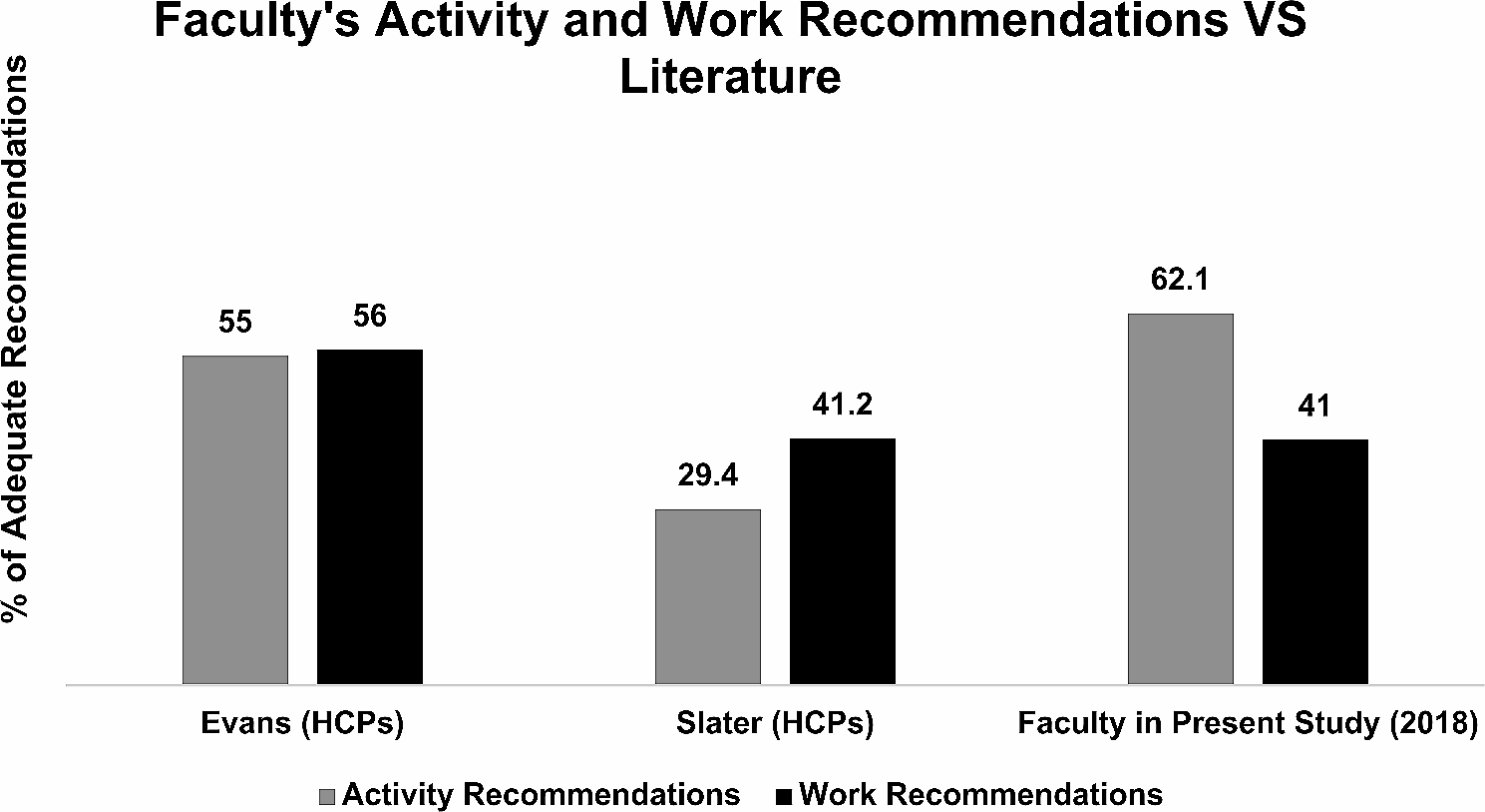
Faculty’s activity and work recommendations vs. literature [33, 34]. HCP: Healthcare providers. Gray shading represents the mean percentage of faculty and professionals that gave adequate activity recommendations. Black shading represents the mean percentage of faculty and professionals that gave adequate work recommendations

There is currently no evidence to suggest a “gold standard” HC-PAIRS score for students, faculty, and/or professionals. However, prior studies have assessed various student and health professionals’ HC-PAIRS scores and activity/work recommendations for patients with PLBP (Tables 3 and 4; Figs. 4 and 5). Results from these prior studies provide helpful context for interpreting scores from our present study. Both student and faculty mean HC-PAIRS scores were higher (indicating stronger beliefs that LBP justifies disability and activity limitation) than those of their counterparts from similar studies in the literature [18, 26, 29–32]. Furthermore, students less frequently gave “adequate” activity and work recommendations [26, 29], while faculty provided similar or more frequent “adequate” recommendations when compared to other licensed clinical professionals [33, 34].

**Table 3:** Mean HC-PAIRS scores from students compared to similar studies in the literature

Paper	Mean Scores	Population
Briggs [29]	3.45	Australian Chiropractic Students (Final Year)
Briggs [29]	3.55	Australian Medical Doctor Students (Final Year)
Latimer [22]	3.47	Australian Physical Therapy Students (3rd-4th Year)
Carroll [30]	4.18	Mixed Health Care Professional Students
Present Study	4.41	Chiropractic Students (Years 1–3)

HC-PAIRS: Healthcare Providers Pain and Impairment Relationship Scale

**Table 4:** Mean HC-PAIRS scores from faculty compared to similar studies in the literature

Paper	Mean Scores	Population
Louw [31]	3.36	Physicians
Louw [31]	3.35	Physical Therapists
Rainville [18]	2.53	Functional Restoration Providers
Caner Aksoy [32]	3.52	Physical Therapists
Domenech [26]	4.26	Family Physicians
Present Study	3.66	Chiropractic Program Faculty

HC-PAIRS: Healthcare Providers Pain and Impairment Relationship Scale

Beliefs that PLBP justifies disability and limitation of activities are inconsistent with current best practices for management of LBP [27, 35, 36]. Current CPGs highlight the importance of encouraging early return to normal daily activities and work-related tasks for patients experiencing back pain [27, 35, 36]. Therefore, the results of our study demonstrate that students and faculty at a single chiropractic college may hold beliefs about patients with PLBP and provide recommendations for these patients that are incongruent with CPGs. Changes in HC-PAIRS and clinical vignette scores toward more CPG-congruent beliefs and recommendations could potentially, in theory, be associated with improved management and outcomes of PLBP patients [18]. In addition, chiropractic college curricula and continuing education courses that promote guideline-adherent beliefs and recommendations regarding PLBP patients could potentially have an impact on clinician behavior [6, 7]. As such, chiropractic colleges could consider early and consistent exposure to evidence-based resources and experiences that promote guideline-congruent beliefs and recommendations with respect to the management of PLBP patients.

Future attempts to change measured attitudes, beliefs, and recommendations via educational interventions could be guided by interventions previously described in the literature. Studies including students from several healthcare disciplines have employed educational interventions on one or more of the following topics with favorable outcomes on attitudes, beliefs, and recommendations for patients with PLBP: pain neuroscience education, the BPS model of pain, identifying yellow flags, giving activity/work recommendations according to CPGs, and using drawings, stories and metaphors to make sense of pain [26, 34, 37, 38]. However, to date, we are not aware of existing literature that suggests what magnitude of change in HC-PAIRS or clinical vignette scores signifies a meaningful change in clinical behavior or improvement in patient outcomes. Future work involving the HC-PAIRS and similar outcome measures should strive to elucidate the relationship between a change in scores and subsequent clinical behaviors and outcomes.

### Limitations

The generalizability of the study is limited, as these data only reflect the results from one chiropractic college. No evidence currently exists synthesizing HC-PAIRS scores for students, faculty, and health professionals. As such, future research in this area would provide helpful context in interpreting HC-PAIRS scores.

## Conclusion

This study assessed the attitudes and beliefs of students and faculty of a single chiropractic college regarding patients with PLBP. Student and faculty scores regarding attitudes and beliefs, and students’ activity/work recommendations were found to be dissimilar to other students and health professionals in prior studies and less congruent with CPG recommendations. This study also found that lower HC-PAIRS scores (indicating higher functional expectations for patients with PLBP) increased the odds of students providing “adequate” activity and work recommendations to patients with PLBP. Results from this study may help guide future research, inform chiropractic college curricula, and augment chiropractic postgraduate education curricula in the management of PLBP.

### Electronic supplementary material

Below is the link to the electronic supplementary material.

Supplementary Material 1

Supplementary Material 2

## Data Availability

The data set is available upon request to the authors.

## Abbreviations

US: United States
LBP: Low back pain
PLBP: Persistent low back pain
HCP: Healthcare providers
HC-PAIRS: Health Care Providers’ Pain and Impairment Relationship Scale
GPA: Grade point average
QR code: Quick response code
CPG: Clinical practice guideline
