# Supplementary material for: Attitudes, beliefs, and recommendations for persistent low back pain patients: cross-sectional surveys of students and faculty at a chiropractic college
Source: Chiropr Man Therap. 2024 Feb 29;32:7. doi: 10.1186/s12998-024-00530-7 (PMC10905815; doi:10.1186/s12998-024-00530-7)
Supplement: Supplementary file 1 — Supplementary Material 1 [file 12998_2024_530_MOESM1_ESM.docx]

Rainville J, Bagnall D, Phalen L. Health care providers’ attitudes and beliefs about functional impairments and chronic back pain. Clin J Pain. 1995 Dec;11(4):287-95.

1. Chronic back pain patients can still be expected to fulfill work and family responsibilities despite pain.

| 1 | 2 | 3 | 4 | 5 | 6 | 7 |
| --- | --- | --- | --- | --- | --- | --- |
| Completely Disagree | Disagree | Disagree Somewhat | Neutral | Agree Somewhat | Agree | Completely Agree |

1. An increase in pain is an indicator that a chronic back pain patient should stop what he is doing until the pain decreases.

| 1 | 2 | 3 | 4 | 5 | 6 | 7 |
| --- | --- | --- | --- | --- | --- | --- |
| Completely Disagree | Disagree | Disagree Somewhat | Neutral | Agree Somewhat | Agree | Completely Agree |

1. Chronic back pain patients cannot go about normal life activities when they are in pain

| 1 | 2 | 3 | 4 | 5 | 6 | 7 |
| --- | --- | --- | --- | --- | --- | --- |
| Completely Disagree | Disagree | Disagree Somewhat | Neutral | Agree Somewhat | Agree | Completely Agree |

1. If their pain would go away, chronic back pain patients would be every bit as active as they used to be.

| 1 | 2 | 3 | 4 | 5 | 6 | 7 |
| --- | --- | --- | --- | --- | --- | --- |
| Completely Disagree | Disagree | Disagree Somewhat | Neutral | Agree Somewhat | Agree | Completely Agree |

1. Chronic back pain patients have the same benefits as the handicapped because of their chronic pain problem.

| 1 | 2 | 3 | 4 | 5 | 6 | 7 |
| --- | --- | --- | --- | --- | --- | --- |
| Completely Disagree | Disagree | Disagree Somewhat | Neutral | Agree Somewhat | Agree | Completely Agree |

1. Chronic back pain patients owe it to themselves and those around them to perform their usual activities even when their pain is bad.

| 1 | 2 | 3 | 4 | 5 | 6 | 7 |
| --- | --- | --- | --- | --- | --- | --- |
| Completely Disagree | Disagree | Disagree Somewhat | Neutral | Agree Somewhat | Agree | Completely Agree |

1. Most people expect too much of chronic back pain patients, given their pain.

| 1 | 2 | 3 | 4 | 5 | 6 | 7 |
| --- | --- | --- | --- | --- | --- | --- |
| Completely Disagree | Disagree | Disagree Somewhat | Neutral | Agree Somewhat | Agree | Completely Agree |

1. Chronic back pain patients have to be careful not to do anything that might make their pain worse.

| 1 | 2 | 3 | 4 | 5 | 6 | 7 |
| --- | --- | --- | --- | --- | --- | --- |
| Completely Disagree | Disagree | Disagree Somewhat | Neutral | Agree Somewhat | Agree | Completely Agree |

1. As long as they are in pain, chronic back pain patients will never be able to live as well as they did before.

| 1 | 2 | 3 | 4 | 5 | 6 | 7 |
| --- | --- | --- | --- | --- | --- | --- |
| Completely Disagree | Disagree | Disagree Somewhat | Neutral | Agree Somewhat | Agree | Completely Agree |

1. When their pain gets worse, chronic back pain patients find it very hard to concentrate on anything else.

| 1 | 2 | 3 | 4 | 5 | 6 | 7 |
| --- | --- | --- | --- | --- | --- | --- |
| Completely Disagree | Disagree | Disagree Somewhat | Neutral | Agree Somewhat | Agree | Completely Agree |

1. Chronic back pain patients have to accept that they are disabled persons, due to their chronic pain.

| 1 | 2 | 3 | 4 | 5 | 6 | 7 |
| --- | --- | --- | --- | --- | --- | --- |
| Completely Disagree | Disagree | Disagree Somewhat | Neutral | Agree Somewhat | Agree | Completely Agree |

1. There is no way that chronic back pain patients can return to doing the things that they used to do unless they first find a cure for their pain.

| 1 | 2 | 3 | 4 | 5 | 6 | 7 |
| --- | --- | --- | --- | --- | --- | --- |
| Completely Disagree | Disagree | Disagree Somewhat | Neutral | Agree Somewhat | Agree | Completely Agree |

1. Chronic back pain patients find themselves frequently thinking about their pain and what it has done to their life.

| 1 | 2 | 3 | 4 | 5 | 6 | 7 |
| --- | --- | --- | --- | --- | --- | --- |
| Completely Disagree | Disagree | Disagree Somewhat | Neutral | Agree Somewhat | Agree | Completely Agree |

1. Even though their pain is always there, chronic back pain patients often don’t notice it at all when they are keeping themselves busy.

| 1 | 2 | 3 | 4 | 5 | 6 | 7 |
| --- | --- | --- | --- | --- | --- | --- |
| Completely Disagree | Disagree | Disagree Somewhat | Neutral | Agree Somewhat | Agree | Completely Agree |

1. All of chronic back pain patients’ problems would be solved if their pain would go away.

| 1 | 2 | 3 | 4 | 5 | 6 | 7 |
| --- | --- | --- | --- | --- | --- | --- |
| Completely Disagree | Disagree | Disagree Somewhat | Neutral | Agree Somewhat | Agree | Completely Agree |
