# Supplementary material for: Attitudes, beliefs, and recommendations for persistent low back pain patients: cross-sectional surveys of students and faculty at a chiropractic college
Source: Chiropr Man Therap. 2024 Feb 29;32:7. doi: 10.1186/s12998-024-00530-7 (PMC10905815; doi:10.1186/s12998-024-00530-7)

Rainville J, Carlson N, Polatin P, Gatchel RJ, Indahl A. Exploration of physicians’ recommendations for activities in chronic low back pain. Spine. 2000;25:2210–20.


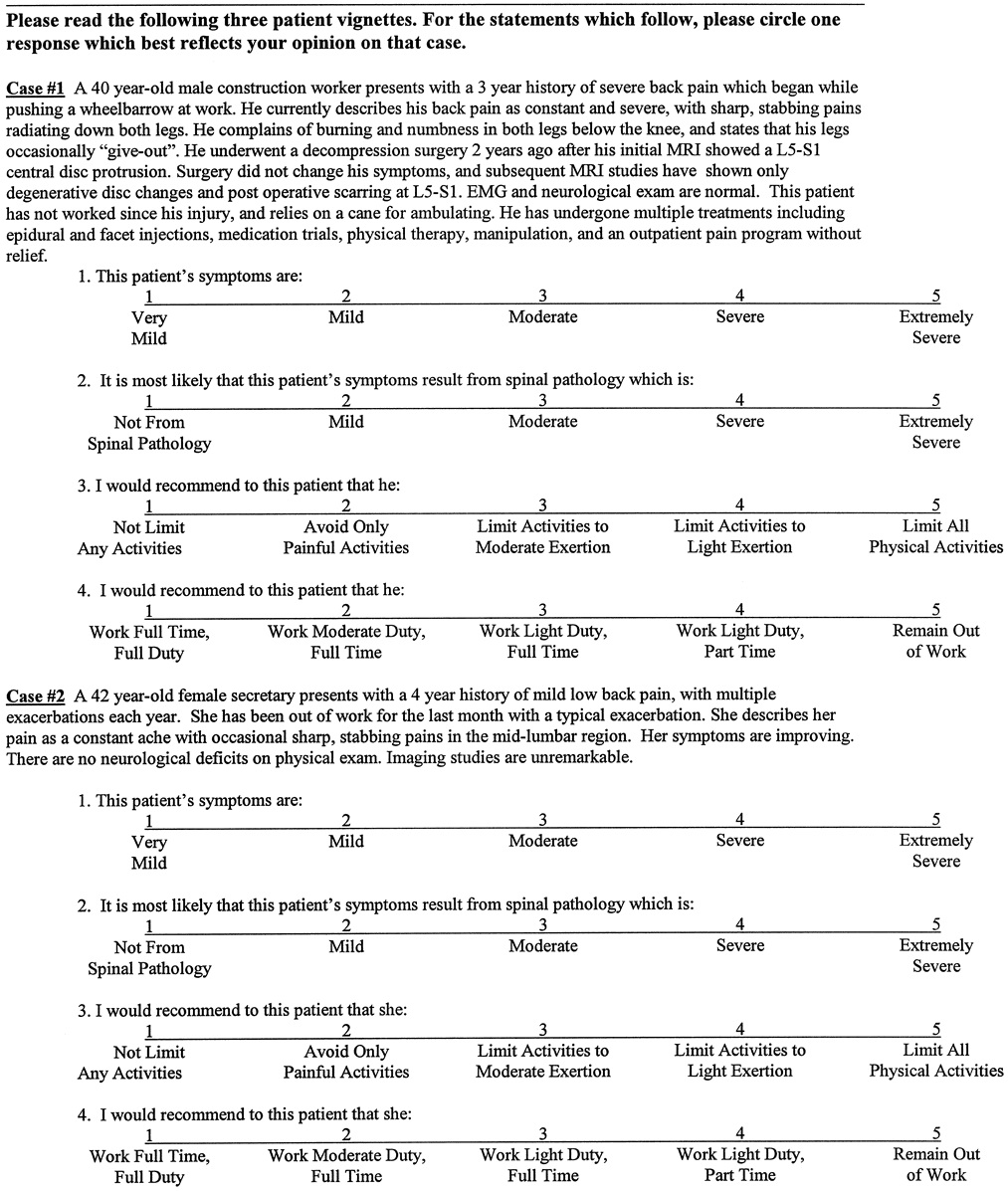


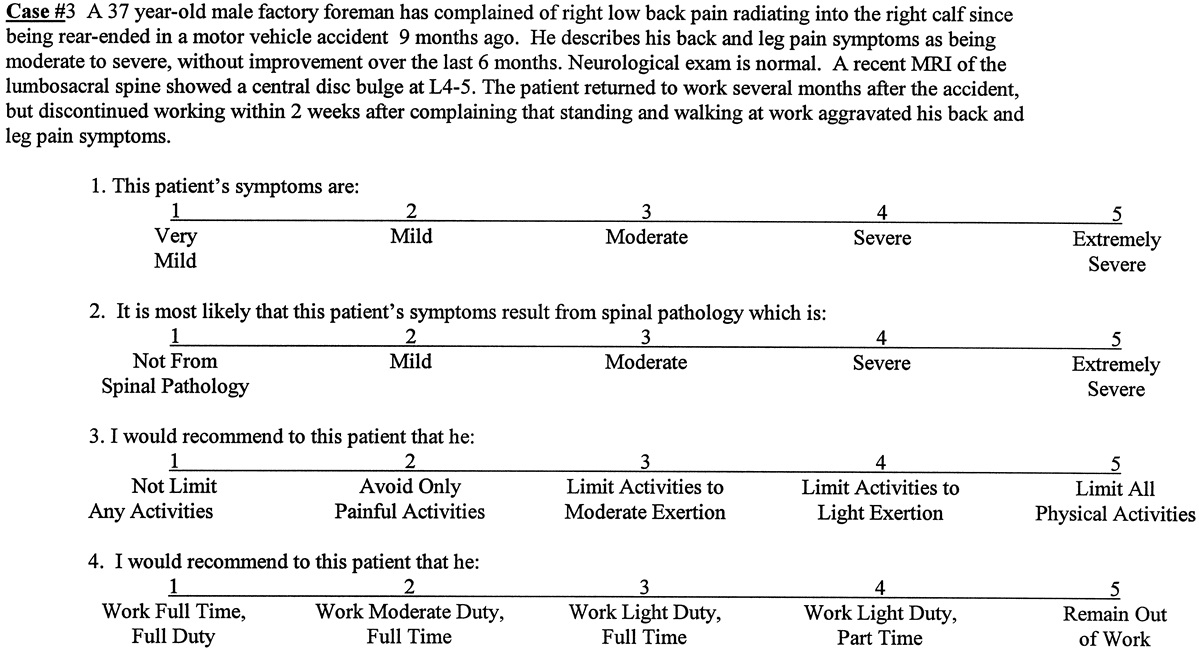

Supplement: Supplementary file 2 — Supplementary Material 2 [file 12998_2024_530_MOESM2_ESM.docx]
